# Improved bioavailability and anti-nephrotoxicity efficacy of polydatin on cisplatin-induced AKI *via* a dual-targeting fucoidan delivery system

**DOI:** 10.1016/j.ijpx.2025.100422

**Published:** 2025-10-18

**Authors:** Yinghan Wang, Shichao Liu, Feikai Zhu, Xuefei Wang, Hanyu Wang, Lin Long, Jun Xiao, Chuanlong Guo

**Affiliations:** a College of Chemical Engineering, Qingdao University of Science and Technology, Qingdao 266042, China; b Department of Oncology, Shandong Provincial Key Medical and Health Discipline, Qingdao Central Hospital, University of Health and Rehabilitation Sciences, Qingdao 266300, China; c College of Clinical Medicain, Jining Medical University, Jining 272067, China; d Oncology Center I Department, Qingdao Hiser Hospital Affiliated of Qingdao University (Qingdao Traditional Chinese Medicine Hospital), Qingdao 266000, China

**Keywords:** Acute kidney injury, Cisplatin, Endoplasmic reticulum stress, cGAS STING pathway, Delivery system

## Abstract

Acute kidney injury (AKI) is a common and serious complication in clinical practice, especially when using chemotherapy drug cisplatin, which severely limits its anti-cancer efficacy. This study developed a novel nano delivery system (Fu-4-PBA/Po NPs) that combines fucoidan (Fu), endoplasmic reticulum stress (ERS) inhibitor 4-phenylbutyric acid (4-PBA), and natural antioxidant Polydatin (Po) to simultaneously alleviate cisplatin induced oxidative stress and ERS. The optimized Fu-4-PBA/Po NPs showed an average size of 102 ± 3.46 nm, a zeta potential of −16.5 ± 0.49 mV, and a high drug loading capacity of 10.34 ± 0.6 %. They exhibited excellent stability and a sustained release profile *in vitro*. *In vitro* results showed that treatment with Fu-4-PBA/Po NPs led to a marked increase in cell survival by 34.73 ± 3.54 percentage points compared to the model group, which had a survival rate of 45.97 ± 2.76 %. Furthermore, Fu-4-PBA/Po NPs effectively inhibited the accumulation of reactive oxygen species (ROS), mitochondrial membrane potential decline, and calcium ion release by targeting P-selectin and endoplasmic reticulum (ER) at the site of inflammation, and significantly alleviate cisplatin induced cell apoptosis. Mechanism studies showed that nanocomposites alleviate ERS by inhibiting the PERK-eIF2α-ATF4-CHOP pathway, while blocking the activation of the cGAS-STING pathway, thereby reducing DNA damage and inflammatory response. Pharmacokinetic studies showed that the peak concentration (Cmax) of the nanoparticles reached 2.2 times that of free Po, and the area under the curve (AUC) increased by 2 times. In animal studies, administration of Fu-4-PBA/Po NPs at a dose of 100 mg/kg notably ameliorated renal function, as indicated by reductions in serum creatinine (SCr) and blood urea nitrogen (BUN) levels by 84.3 % and 62.4 %, respectively, and also improved kidney histopathology. This study provided a dual targeted nano-delivery strategy for cisplatin induced AKI therapy, which has important clinical application potential.

## Graphical abstract

**Figure:**
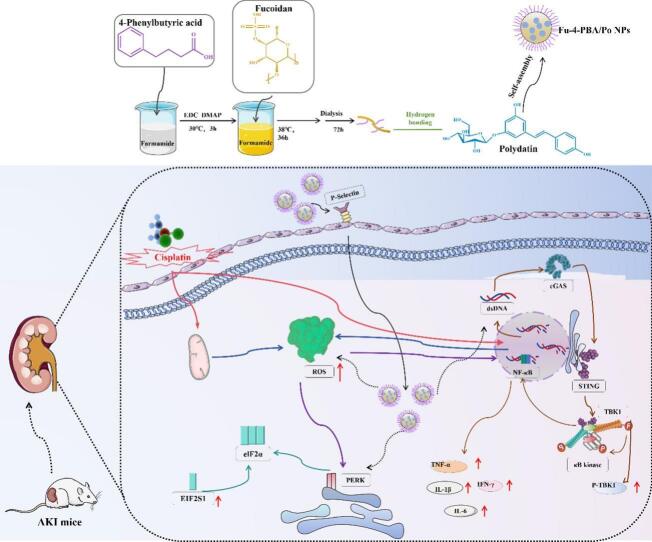

## 1 Introduction

Owing to the aging population, evolving dietary habits, and other relevant factors, acute kidney injury (AKI) has emerged as a critical global public health concern, impacting over 10 million patients each year.(Geo et al., 2022; Nie et al., 2022) AKI is typically marked by a sudden deterioration of kidney function within a short span of hours to days. This results in a swift rise in serum creatinine (Scr) and blood urea nitrogen (BUN) levels, accompanied by a reduction in urine output.(Ba et al., 2024) In numerous AKI patients, renal function fails to achieve complete recovery, leaving these patients at risk of long-term morbidity and mortality. Furthermore, AKI notably increases the likelihood of patients developing cardiovascular diseases, chronic kidney disease, and even end-stage renal disease.(Wang et al., 2023) Clinically, AKI is a complex syndrome with diverse etiologies. It can be caused by generalized or localized ischemic damage to the kidneys due to surgery, sepsis, trauma, as well as toxic reactions to medications including antibiotics and chemotherapeutic agents. For example, the administration of the chemotherapeutic drug cisplatin may result in AKI.(McSweeney et al., 2021).

Cisplatin ranks among the most potent anti – cancer chemotherapy agents and is widely employed in the treatment of a broad range of solid tumors.(Zhou et al., 2022b) However, the complete anti - cancer potential of cisplatin remains untapped, mainly due to the toxicities associated with the treatment. Among these toxicities, AKI is especially conspicuous, being identified in roughly 33 % of patients.(Williams et al., 2021) Once AKI develops in these patients, it may lead to the termination of chemotherapy or a shift to less effective chemotherapeutic regimens. Therefore, the development of novel strategies to mitigate cisplatin - induced AKI has the potential to significantly improve the outcomes of cancer treatment and enhance the quality of life of cancer survivors.

Oxidative stress has been firmly established as a major pathogenic factor in Cisplatin-induced AKI.(Wang et al., 2021) In light of this, functional nanostructures that possess strong antioxidant activity and are characterized by high biocompatibility, low toxicity, and kidney - targeting specificity have piqued the interest of both the nanotechnology and medical fields, holding great potential for the protection and treatment of kidney injury.(Wei et al., 2023) Antioxidant natural compounds, such as those derived from traditional Chinese medicines, present promising outlooks for the prevention and treatment of cisplatin - induced AKI. These compounds are distinguished by their high efficacy, low toxicity, and multi - targeting capabilities.(Cai et al., 2022; Kumar, 2023; Yan et al., 2023) Take Polydatin (Po), for example. This natural active small molecule, extracted from the roots of *Polygonum cuspidatum* (Polygonaceae), possesses potent antioxidant activity. Its molecular structure, characterized by conjugated double bonds, endows it with this remarkable antioxidative property.(Du et al., 2013) Through diverse molecular mechanisms, Po exerts notable protective and curative effects on diseases associated with oxidative stress.(Tang et al., 2022) Furthermore, Po also demonstrates strong anti - inflammatory activity, which can play an auxiliary role in relieving kidney injury.(Gao et al., 2020; Söhretoglu et al., 2018; Yang et al., 2024b) However, the current clinical application of Po has some limitations, mainly due to its poor water solubility and low oral bioavailability (2.9 %),(Chen et al., 2020; Liu et al., 2015) The development of an alternative drug delivery system is highly desirable.

Following the onset of AKI, endoplasmic reticulum stress (ERS) is initiated.(Yang et al., 2024a) First, the pathways involved in the unfolded protein response (UPR) are activated. This includes protein kinase RNA - like endoplasmic reticulum kinase (PERK), inositol - requiring enzyme 1 (IRE1), and activating transcription factor 6 (ATF6).(Huang et al., 2020) Subsequently, ERS induces two crucial forms of cell death. One is ferritinophagy - mediated ferroptosis, and the other is caspase 1, interleukin (IL) - 1β, and IL - 18 - mediated pyroptosis. These processes ultimately promote the progression of AKI.(He et al., 2021b) ERS also lead to activation of the cGAS-STING pathway. The activated STING can promote the phosphorylation and activation of PERK, and induce the expression of type I interferon (IFN-I) and pro-inflammatory factors through the TBK1 signaling pathway, further exacerbating inflammation and cell damage.(Zhang et al., 2022; Zhang et al., 2024) Therefore, if antioxidant natural compounds can mitigate the cisplatin - induced ERS, they can alleviate AKI more rapidly and effectively.

In this paper, to develop a novel drug delivery system capable of efficiently alleviating both oxidative stress and ERS simultaneously, we employed fucoidan (Fu) as the drug carrier. After chemically modifying it by attaching 4 - phenylbutyric acid (4-PBA), we loaded the antioxidant natural small molecule, polydatin, onto it. As a result, a Fu-4-PBA/Po nanocomplex that is stable and uniformly dispersed in aqueous solutions was successfully obtained. 4-PBA serves as an ERS inhibitor, which can modulate the restoration of ER homeostasis and address multiple related pathological conditions.(Hong et al., 2018; Zhao et al., 2024) Fu, a natural biomacromolecule, shares certain similarities in chemical structure and composition with the organism's own polysaccharide substances. This characteristic reduces the likelihood of triggering strong immune rejection reactions upon entering the organism (Fitton et al., 2015; Pozharitskaya et al., 2018). Instead, it can readily adapt to the organism's internal tissues and cells, thereby ensuring the safe delivery of drugs. Furthermore, the numerous functional groups in fucoidan's molecular structure offer the potential for functional chemical modification. Through cell and animal experiments, in - depth investigations were carried out into the structure, function, and mechanism of the Fu-4-PBA/Po nanocomposites for alleviating cisplatin - induced AKI.

## 2 Experimental

### 2.1 Materials

Fucoidan (C_6_H_10_O_7_S)_n_ (MW: 15989.41, purity ≥98 %)， extracted from brown seaweed were purchased from Shanghai Aladdin Biochemical Technology Co., Ltd. (Shanghai, China). Polydatin (C_20_H_22_O_8_) (MW: 390.39, purity ≥95 %), Rhodamine 123 (purity≥99 %) were purchased from Shanghai Macklin Biochemical Co., Ltd. (Shanghai, China). 4-Phenylbutyric acid (4-PBA, C_10_H_12_O_2_, MW: 164.2, purity ≥99 %), was purchased from Shanghai Aladdin Biochemical Technology Co., Ltd. (Shanghai, China). 4-Dimethylaminopyridine (C_7_H_10_N_2_) (MW: 122.17, purity≥99 %) was purchased from aladdin (Shanghai, China). N-(3-Dimethylaminopropyl)-N′-ethylcarbodiimide hydrochloride (C_8_H_18_ClN_3_), (MW: 191.7, purity ≥98 %) was purchased from Shanghai Macklin Biochemical Co., Ltd. (Shanghai, China). Fluo-4 AM was purchased from Wuhan Severus Biotechnology Co., Ltd. (Wuhan, China). Cisplatin (Pt(NH_3_)_2_Cl_2_) (MW: 300.05, purity≥99 %) was purchased from Shanghai Xian Ding Biotechnology Co., Ltd. (Shanghai, China). Human umbilical vein endothelial cells (HUVEC) and mouse kidney cells (HK−2) were purchased from the BeNa Culture(Henan, China).phosphate-buffered saline (PBS), Agarose, 4 % Para­formaldehyde, and cell culture medium RPMI-1640, were received from Solarbio® Life Science (Beijing, China). 3-(4,5-dimethylthiazol-2-yl)-2,5-diphenyltetrazolium bromide (MTT), Hoechst 33258, and DCFH-DA were purchased from Beyotime Biotechnology (Shanghai, China). and all other reagents were of analytical grade. 2 × SYBR Green qPCR Master Mix (High ROX) was purchased from servicebio (Wuhan, China). Antibodies used in this study were listed in Table S1.

### 2.2 Preparation of Fu-4-PBA/Po NPs

The preparation of Fu-4-PBA/Po nanoparticles was carried out using the anti-solvent method. Fu-4-PBA was obtained by modifying 4-PBA onto Fu. First, 67 mg of 4-PBA was dissolved in 70 ml of formamide, and the carboxyl groups were activated with 28 mg of EDC and 18 mg of DAMP at 30 °C for 3 h. Then, 500 mg of Fu was dissolved in 30 ml of formamide at 50 °C. After the activation was complete, the two solutions were mixed and allowed to react fully at 38 °C for 48 h. Subsequently, the reaction mixture was dialyzed for 36 h (3000 Da) to remove the activators and any unreacted compounds from the system. The obtained product was characterized by ^1^H NMR and FITR(Gao et al., 2022a).

For the preparation of NPs, 70 mg of Fu-4-PBA was dissolved in 8.5 mL of deionized water and stirred at 500 rpm at room temperature until completely dissolved. Meanwhile, 10 mg of Po was dissolved in 1.5 mL of ethanol and sonicated until fully dissolved. The Po-containing ethanol solution was slowly dripped into the Fu-4-PBA aqueous solution under continuous sonication to obtain Fu-4-PBA/Po nanoparticles. Unreacted Fu-4-PBA and Po were removed by high-speed centrifugation (12,000 rpm, 20 min).

The determination of Po content in Fu-4-PBA/Po NPs was performed using a UV spectrophotometer by measuring the wavelength of Po. Briefly, 1 mL of the freshly prepared Fu-4-PBA NPs was divided into two groups: one group was unfiltered (Solution A), and the other group was filtered through a 0.22 μm filter (Solution B). The absorbance values were measured at 317 nm using a spectrophotometer. Finally, the Po content was estimated based on a standard calibration curve. The encapsulation efficiency (EE) and drug loading (DL) of Po were calculated using the following formulas.(Gao et al., 2023).

EE was calculated according to the following equation:

$$\mathrm{EE}\;\left(\%\right)=\frac{\mathrm{Po}\;\text{in soltion}\;B}{\mathrm{Po}\;\text{in solution}\;A}\times 100\%.$$

DL capacity was calculated accordingly to the following equation:

$\mathrm{DL}\;\left(\%\right)==\frac{\text{Encapsulated}\;\mathrm{Po}\;\left(\mathrm{mg}\right)}{\text{Total nanoparticles}\;\left(\mathrm{mg}\right)}\times 100\%$

### 2.3 Characterization of Fu-4-PBA/Po NPs

#### 2.3.1 Particle size

The particle size distribution of the samples was measured using a dynamic light scattering (DLS) instrument. (ZetaPALS; Malvern In-struments Co., Ltd.) as described previously.(Gao et al., 2023)

#### 2.3.2 Transmission Electron Microscopy (TEM)

The nanostructure of the Fu-4-PBA/Po NPs was observed by transmission electron microscopy (TEM, Hitachi H-7650, Japan).(Dong et al., 2023)

#### 2.3.3 Stability of Fu-4-PBA/Po NPs

The stability study of Fu-4-PBA/Po NPs was conducted over a period of 2 months. Briefly, Fu-4-PBA/Po NPs were prepared and stored at room temperature in deionized water. The particle size and zeta-potential were measured every week.

#### 2.3.4 Hemolysis assay

Briefly, the experiment included the following groups: positive control, negative control, Fu, 4-PBA, Fu-4-PBA, Po, physical mixture, and NPs groups. A fixed amount of each sample was added to physiological saline containing 0.1 ml of an anticoagulant and incubated at 37 °C for 1 h. Then, 1.5 ml of the solution from each group was collected and photographed against a white background. Additionally, a quantitative aliquot was taken from each sample to measure the absorbance at 545 nm. All experiments were performed in triplicate.

#### 2.3.5 Drug release study

The *in vitro* release behavior of Fu-4-PBA/Po nanoparticles was evaluated as follows: nanoparticles (1 mg/mL, 5 mL) were sealed in a dialysis bag (molecular weight cutoff: 3500 Da) and immersed in 40 mL of simulated gastric fluid (pH 2.0) containing 10 g/L pepsin. The system was incubated at 37 °C under shaking at 100 rpm. Every 30 min, 100 μL of release medium was sampled, and its absorbance at 317 nm was measured immediately. An equal volume of fresh simulated gastric fluid was replenished after each sampling. After 2 h, the dialysis bag was transferred to 40 mL of simulated intestinal fluid (pH 6.8) containing 10 g/L trypsin, and the release study was continued under the same conditions with sampling at 30-min intervals. The total release duration was 6 h.

### 2.4 *In vitro* antioxidant assay

The antioxidant capacity of Fu-4-PBA NPs was evaluated using the FRAP (Ferric Reducing Antioxidant Power) assay(Liu et al., 2022).

### 2.5 Cell viability assay

Human renal tubular epithelial cells (HK-2) were purchased from the BeNa Culture Collection (Henan, China). Cells were cultured in RPMI-1640 medium supplemented with high glucose and 10 % (*v*/v) FBS, incubated in a humidified 5 % CO_2_ incubator at 37 °C.

HK-2 cells were seeded in 96-well plates and incubated for 24 h. Cells were co-treated with blank medium, Fu-4-PBA, various concentrations of Po or Fu-4-PBA/Po NPs (10 μg/mL) and cisplatin (20 μM) for 24 h. The culture medium was removed and MTT (0.5 mg/mL) was added for further treatment for 4 h. After the supernatant was discarded, 150 μL of DMSO was added and shaken for 10 min. Finally, the absorbance at 490 nm was measured and the inhibition rate was calculated.

To evaluate cell safety, HK-2 cells were seeded into a 96-well plate and incubated for 24 h. Subsequently, blank culture medium, Fu-4-PBA, and various concentrations of Fu-4-PBA/Po nanoparticles were added. After incubating for 48 h, cell viability was measured by MTT assay, as mentioned above.

### 2.6 Cell fluorescence detection experiments and Flow Cytometric Analysis

HK-2 cells were seeded in 24-well plates at a density of 1 × 10^5^ cells per well and cultured for 24 h in a 5 % CO₂ incubator at 37 °C to facilitate adhesion. The cells were then treated for 24 h with the following: blank culture medium, Fu-4-PBA, polydatin (Po, 10 μg/mL), Fu-4-PBA/Po nanoparticles (10 μg/mL), and cisplatin (20 μM). After treatment, the cells were washed twice with PBS.

Three distinct fluorescent probes were used to evaluate cellular stress responses:

• Reactive oxygen species (ROS) levels were detected using DCFH-DA;
• Mitochondrial membrane potential was assessed using Rhodamine 123;
• Intracellular calcium flux was measured using Fluo-4 AM.

Fluorescence images were acquired immediately after staining using a fluorescence microscope. Quantitative analysis of fluorescence intensity was performed with ImageJ software. All experiments were independently repeated at least three times.

For flow cytometry, HK-2 cells were plated in 6-well plates at 5 × 10^5^ cells per well and incubated under the same conditions for 24 h. Cells were treated identically as above, then harvested, washed twice with PBS, and stained with the corresponding fluorescent dyes. Analysis was carried out using a flow cytometer.

For apoptosis assay, HK-2 cells (5 × 10^5^ per well in 6-well plates) were cultured for 24 h and then treated for 24 h with blank medium, Fu-4-PBA, Po (10 μg/mL), Fu-4-PBA/Po NPs (10 μg/mL), and cisplatin (20 μM). After washing with PBS, cells were trypsinized, collected, and resuspended in ice-cold PBS. Apoptosis was detected using an Annexin V-FITC/PI kit: cells were stained with Annexin V-FITC and PI in binding buffer for 15 min in the dark and analyzed by flow cytometry. Experiments were repeated three times independently.

### 2.7 Co-localization of NPs with the Endoplasmic Reticulum and Uptake Validation

Nanoparticles loaded with coumarin 6 (Cou6) were prepared to evaluate intracellular uptake and localization. Briefly, Fu-4-PBA (70 mg in 8.5 mL deionized water) and Cou6 (10 mg in 1.5 mL ethanol) were separately dissolved. The Cou6 solution was then added dropwise under sonication into the Fu-4-PBA solution. Unincorporated materials were removed by centrifugation (12,000 rpm, 20 min).

Prior to adding nanoparticles, HK-2 cells were stained with an endoplasmic reticulum red probe (*e.g.*, ER-Tracker Red) and incubated at 37 °C in the dark for 30 min. Subsequently, fluorescently labeled nanoparticles (Fu-4-PBA/Cou6) were added to the upper chamber of the Transwell insert to observe the uptake of coumarin 6 by HK-2 cells. The experiment was divided into three groups:

- Group 1: Normal nanoparticles (polydatin replaced with coumarin 6);

- Group 2: Nanoparticles + P-selectin inhibitor;

- Group 3: Free coumarin 6 as the control group.

Fluorescence imaging of each group was performed using a confocal microscope. The fluorescence signals of coumarin 6 (excitation wavelength 488 nm, emission wavelength 505 nm) and the endoplasmic reticulum probe (excitation wavelength 587 nm, emission wavelength 615 nm) were detected separately. The co-localization of nanoparticles with the endoplasmic reticulum and the uptake efficiency of coumarin 6 among the groups were evaluated.

### 2.8 Immunofluorescence Staining of Cells

HK-2 cells were seeded on coverslips in 24-well plates at a density of 1 × 10^5^ cells per well and incubated for 24 h in a 37 °C, 5 % CO_2_ incubator. Subsequently, the cells were co-treated with blank medium, cisplatin + Fu-4-PBA, cisplatin + Po (10 μg/mL), cisplatin + Fu-4-PBA/Po nanoparticles (10 μg/mL), and cisplatin (20 μM) for 24 h. After treatment, the culture medium was removed, and the cells were washed three times with PBS for 5 min each. The cells were fixed with 4 % paraformaldehyde for 15 min, followed by three washes with PBS. Permeabilization was performed using 0.1 % Triton X-100 for 10 min, and the cells were washed three times with PBS. Blocking was carried out with 5 % BSA for 1 h at room temperature. After removing the blocking solution, the cells were incubated with diluted primary antibodies at 4 °C overnight. The next day, the cells were washed three times with PBS and incubated with fluorescence-labeled secondary antibodies in the dark at room temperature for 1 h. After three washes with PBS, the nuclei were stained with Hoechst 33258 for 5 min, followed by three washes with PBS. The coverslips were mounted onto glass slides using an anti-fade mounting medium. Images were captured using a fluorescence microscope, and fluorescence intensity was analyzed using ImageJ software. Each experiment was repeated at least three times.

### 2.9 Western blot assay

HK-2 cells were seeded in 96-well plates and incubated for 24 h. Cells were co-treated with blank medium, Fu-4-PBA, Po (10 μg/mL), Fu-4-PBA/Po NPs (10 μg/mL) and cisplatin (20 μM) for 24 h. Proteins were extracted and separated by SDS-PAGE, transferred to PVDF membrane, and then blocked with skim milk powder for 1 h. The membranes were incubated with primary antibody at 4 °C overnight. After 3-times of washes, the membranes were incubated with HRP-labeled secondary antibody for 2 h. After 3-times of washes, the membranes were ultimately embedded with Pierce ECL Plus Western Blotting substrate and imaged. The in­tensities were analyzed by the Image J.

### 2.10 Animals

Male Kunming mice weighing 20–25 g, SD rats weighing 180–200 g were acquired from Daren Fucheng Experimental Animal Breeding Co., Ltd. They were maintained under standard conditions, with 12-h light/dark cycles, as well as *ad libitum* access to water and food. The animal experiments in this research work complied with the ARRIVE guidelines and were conducted as per the U.K. Animals (Scientific Procedures) Act, 1986, and associated guidelines. The animal study was approved by the Qingdao University of Science and Technology Ethics Committee for Animal Experimentation (Approval Number: QKDLL-2024–57).

### 2.11 *In vivo* pharmacokinetic and Organ distribution study

SD rats were randomly divided into two groups (*n* = 3) and fasted overnight. The animals were orally administered free polydatin (Po) or Fu-4-PBA/Po. Blood samples were collected at time points of 0 min, 15 min, 60 min, 120 min, 240 min, 360 min, 480 min, 600 min, 720 min, 840 min, and 1440 min. Methanol was added to the plasma to completely precipitate proteins and other substances while simultaneously extracting polydatin from the plasma. The samples were stored overnight in a 4 °C refrigerator to ensure complete protein precipitation, resulting in a methanol solution containing polydatin. The supernatant was collected and mixed with an internal standard. For the measurement of polydatin, a C18 column was used with a column temperature of 35 °C, a flow rate of 1.0 ml/min, and a detection wavelength of 306 nm. The mobile phase consisted of acetonitrile-water (23:77). The peak areas of polydatin and the internal standard were measured, and the ratio was calculated and substituted into the standard curve for quantification.

### 2.12 *In vivo* imaging analysis

Fu-4-PBA/polydatin-DIR nanoparticles were prepared for *in vivo* imaging. Briefly, 70 mg of Fu-4-PBA was dissolved in 8.5 mL deionized water under stirring (500 rpm) at room temperature. Simultaneously, 10 mg of polydatin and a stoichiometrically calculated amount of DIR (5.098 μg, corresponding to 5 μM final concentration in 10 mL) were co-dissolved in 1.5 mL ethanol *via* sonication. The ethanol solution was then added dropwise under continuous sonication into the Fu-4-PBA aqueous solution. The resulting nanoparticles were collected by centrifugation (12,000 rpm, 20 min) to remove unincorporated Fu-4-PBA, polydatin, and DIR.

To evaluate the localization of Fu-4-PBA nanocarriers *in vivo*, we pre­pared Fu-4-PBA nanoparticles loaded with DIR dye and administered them to mice by gavage. The distribution of fluorescence was analyzed through an *in vivo* imaging system (IVIS, Lumina XRMS, PerkinElmer, USA). Subsequently, the mice were euthanized with carbon dioxide, and visceral tissues such as heart, liver, spleen, lungs, and kidneys were collected. Fluorescence distribution in different tissues was analyzed using the *in vivo* imaging system.

### 2.13 *In vivo* studies

#### 2.13.1 Animal grouping

The animals were randomly divided into 7 groups as follows:

• blank control group (normal saline);
• cisplatin model group (20 mg/kg, single intraperitoneal injection);
• nanoparticle treatment groups (Fu-4-PBA/Po NPs at 50 or 100 mg/kg + cisplatin);
• polydatin treatment groups (free polydatin at 50 or 100 mg/kg + cisplatin);
• excipient control group (Fu-4-PBA + cisplatin).

All treatments were administered orally 2 h prior to cisplatin injection and continued once daily for four consecutive days. On day 5, all animals were euthanized for terminal sampling. Blood was collected *via* cardiac puncture using EDTA as an anticoagulant for hematological analysis. Serum was separated by centrifugation at 3000 rpm for 15 min for the assessment of renal function markers, including blood urea nitrogen (BUN) and serum creatinine (Scr).

Kidney tissues were processed for further analysis: one portion was fixed in 4 % paraformaldehyde for sectioning and histological evaluation (including tubular injury scoring), and the other was snap-frozen in liquid nitrogen and stored at −80 °C for subsequent molecular biology assays. Animal body weight was monitored throughout the study. The kidney and spleen indices were calculated as the ratio of the organ's wet weight to the final body weight of the mouse, multiplied by 100 %.

#### 2.13.2 Hematoxylin and eosin (H&E) and immunohistochemical assay

The tissues were fixed with 10 % neutral formaldehyde, dehydrated, embedded, and sliced into 8 μm sections. Subsequently, the sections were stained with H&E and photographed under a microscope. Immu­nohistochemical was performed according to our previous reported (Dong et al., 2023). Briefly, following deparaffinization and antigen retrieval, the kidney sections were incubated with primary antibodies against PERK, cGAS, STING, and γH2AX (for detailed antibody information, please refer to Table S1).

#### 2.13.3 Real time fluorescence quantitative reverse transcription (RT-qPCR)

Total RNA in kidney tissues was extracted by TRIzol (Thermo Fisher Scientific, Waltham, MA, USA) and reverse transcribed into cDNA, fol­lowed by quantitative PCR analysis. The primer information was shown in Table S2.

### 2.14 Statistical analysis

Data are presented as the mean ± standard deviation (SD). Statistical analysis was performed using SPSS software. Differences between two groups were analyzed using the Student's *t*-test. For comparisons among three or more groups (*in vitro* and *in vivo*, one-way analysis of variance (ANOVA) was employed, followed by Tukey's honestly significant difference (HSD) post-hoc test for multiple comparisons. A *p*-value of less than 0.05 was considered statistically significant.

## 3 Results and discussion

### 3.1 Preparation and characterization of Fu-4-PBA/Po NPs

**Fig. 1:**
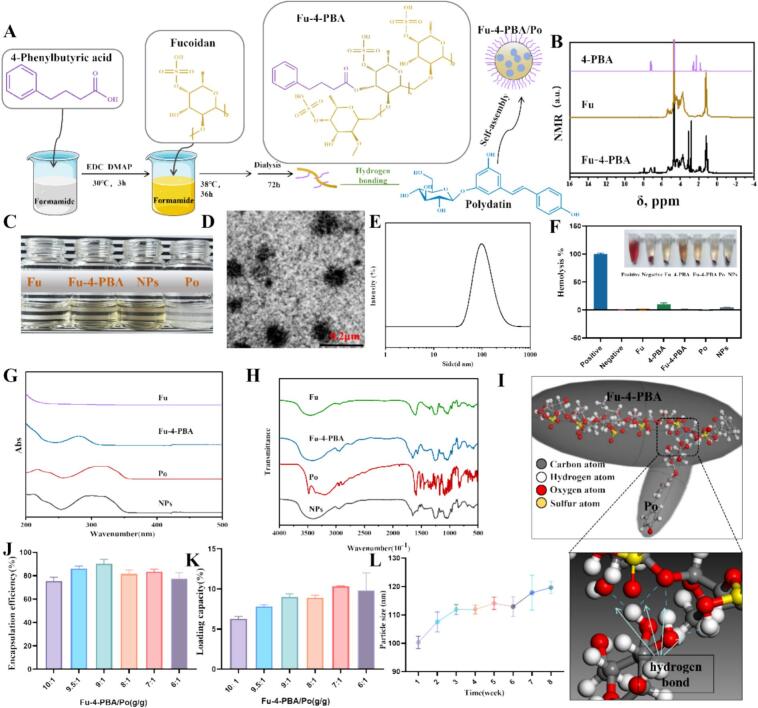
(A) Preparation diagram of Fu-4-PBA/Po NPs. (B) ^1^HNMR spectra of Fu, 4-PBA, and Fu-4-PBA. (C) Solution appearance of Fu, Fu-4-PBA, Fu-4-PBA/Po NPs, and Free Po (The mass ratio of Fu-4-PBA to Po in the nanoparticles was 7:1, with a Po concentration of 1 mg/mL). (D) TEM morphology, (E) Particle size, (F) Hemolysis rate, (G) UV–Vis spectra, (H) FTIR spectra of Fu-4-PBA/Po NPs. (I) Molecular Dynamics of Fu-4-PBA with Po. (J) Encapsulation efficiency and (K) drug loading capacity of Fu-4-PBA/Po NPs at different Fu-4-PBA and Po weight ratios. (L) Stability of Fu-4-PBA/Po NPs at room temperature (with weekly intervals). All experiments were repeated at least three times. Data are presented as the means ± SD.

When cells are stimulated by various internal and external factors, the protein folding function of the ER is affected, leading to the accumulation of unfolded or misfolded proteins in the ER lumen and triggering (ERS). Sustained endoplasmic reticulum stress activates the unfolded protein response (UPR) (Lu et al., 2014; Luchetti et al., 2017). If this stress state cannot be relieved in time, it will lead to cellular dysfunction and even apoptosis. 4-Phenylbutyric acid (4-PBA) is a small-molecule fatty acid that can enter cells and accumulate in the endoplasmic reticulum. By improving the microenvironment of the ER, it promotes the correct folding of proteins, thereby alleviating ERS (He et al., 2021a). To endow nanoparticles with the ability to alleviate ERS, 4-PBA was initially linked to the Fucoidan (Fu) structure *via* an esterification reaction to form Fu-4-PBA (Fig. 1A and 1B). Subsequently, the small-molecule drug Polydatin (Po) was encapsulated within the Fu-4-PBA by means of the anti-solvent method, resulting in the formation of Fu-4-PBA/Po NPs. When serving as a drug carrier, Fu bestows drugs with outstanding biocompatibility, water solubility, and shield drugs from the impacts of the internal environment, thereby extending their half-lives (Fitton et al., 2015; Liu et al., 2025).

As shown in Fig. 1C, when the mass ratio is 7:1, the Fu-4-PBA/Po nanoparticles exhibit excellent dispersibility in aqueous solution. At that time, the aqueous solution was still in a clear and transparent state, without visible coagulation or precipitation, which was significantly different from the turbid state of Po alone. The formation of NPs substantially enhances the water-solubility of Po. The results showed that the solubility of free Po in water was 0.41 ± 0.092 mg/mL (meaning that when 1 mg of Po was added to 1 mL of water, the amount dissolved was 0.41 ± 0.092 mg). After solubilization through a nanoparticle delivery system, its solubility significantly increased to 0.83 ± 0.15 mg/mL, approximately 2.02 times higher than in water. The maximum drug-loading concentration of Fu-4-PBA/Po was 2.5 ± 0.052 mg/mL. This improvement enables Po to be more readily absorbed into the bloodstream through biological membranes. Consequently, both the absorption quantity and rate of Po in the body are increased, and ultimately, its bioavailability is enhanced (Wang et al., 2025b). Fu-4-PBA/Po NPs exhibit a typical nanoparticle morphology (Fig. 1D), with the most frequent particle size being 102 ± 3.46 nm approximately (Fig. 1E). Fu-4-PBA/Po NPs showed no signs of hemolysis (the hemolysis rate is 4.92 % at a concentration of 10 μg/ml NPs), indicates that they possess outstanding blood compatibility, rendering them suitable for biomedical applications (Fig. 1F).

From the UV–visible spectrum in Fig. 1G, it is evident that Fu-4-PBA/Po NPs retain the UV absorption peak characteristic of Po itself. Moreover, the presence of Fu-4-PBA leads to a slight blue-shift of the absorption peak. This phenomenon indicates that Fu-4-PBA/Po NPs do not alter the chemical structure of Po, implying that they are likely to preserve the pharmacological properties of Po. The FTIR spectra showed similar results (Fig. 1H), with no new peaks in the infrared spectra of Fu-4-PBA/Po NPs distinct from Fu and Po alone, indicating that the chemical structure of Po has not been altered.

Po may form Fu-4-PBA/Po NPs by combining with Fu-4-PBA through hydrogen bonding (Fig. 1I) as well as π-π superposition. Upon increasing the initial quantity of Po added, it is observable that the encapsulation efficiency of Po in Fu-4-PBA/Po NPs remains largely constant, stabilizing above 75 % (Fig. 1J). Meanwhile, the drug loading capacity rises in direct proportion to the initial addition of Po (Fig. 1K). This finding validates the affinity and binding capacity between Po and Fu-4-PBA. In addition, Fu-4-PBA/Po NPs demonstrate outstanding stability in the aqueous phase, and its encapsulation efficiency and drug loading remained stable (Fig. S1). The particle size of the NPs remains stable at room temperature for approximately eight weeks, not exceeding 120 nm (Fig. 1L). The zeta-potential also remained stable, maintaining around −16 mV for 8 weeks (Fig. S2). *In vitro* release studies revealed distinct profiles for the two loaded components. The physically encapsulated Po exhibited a relatively rapid yet sustained release behavior. Approximately 40 % of Po was released within 2 h in simulated gastric fluid (SGF, pH 2.0). After transferring to simulated intestinal fluid (SIF, pH 6.8), the cumulative release of Po continued, reaching approximately 80 % over 6 h. This release pattern supported oral delivery by limiting early release in the stomach and promoting gradual intestinal release, the major site of drug absorption. Notably, 4-PBA, which was conjugated *via* an ester bond, demonstrated a pH-dependent and enzyme-triggered release profile. Minimal release (<10 %) occurred in SGF, while significantly increased and sustained release was observed in SIF, attributable to trypsin-mediated hydrolysis and higher pH (Fig. S3). It is important to note that the NPs' stability and controlled release profile under harsh digestive conditions were further verified by *in vitro* release studies simulating the extreme gastrointestinal environment (highly acidic gastric fluid and enzyme-rich intestinal fluid). Furthermore, pharmacokinetic studies revealed that the drug concentration peaked at 2 h after the administration of the NPs (Fig. 7E). These results confirm the controlled (“smart”) release capacity of the nanoparticle system, which protects 4-PBA from the acidic gastric environment and enables targeted intestinal release.

### 3.2 Fu-4-PBA/Po NPs protect against cisplatin induced HK-2 cell injury

**Fig. 2:**
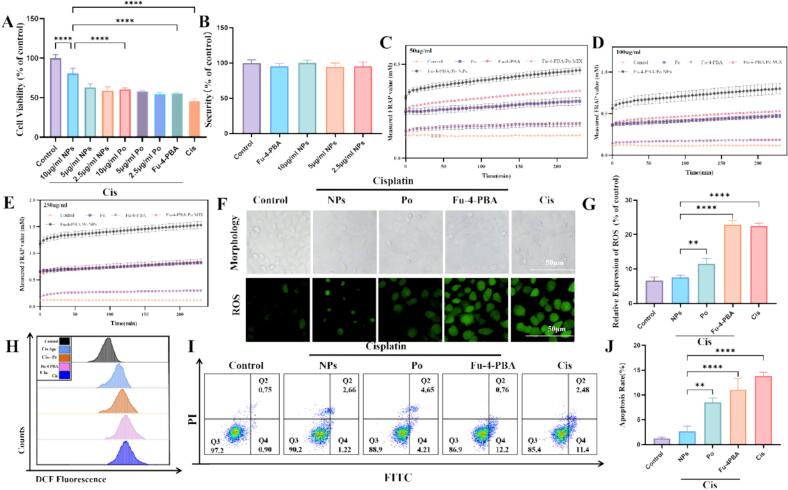
The kidney protective effect of Fu-4-PBA/Po NPs on HK-2 cells. (A) The effect of drugs on the proliferation of HK-2 cells was detected using MTT assay. (B) The safety of NPs at different concentrations. (C-E) The antioxidant capacity of Control, Po, Fu-4-PBA, MIX, and NPs was evaluated at various concentrations. (F, G) The cell morphology of HK-2 cells after treatment with different drugs, and reactive oxygen species (ROS) were detected by DCFH-DA staining. (H) Detection of reactive oxygen species (ROS) by DCFH-DA staining combined with flow cytometry. (I, J) Detection of cell apoptosis by FITC/PI double staining combined with flow cytometry, and quantitative analysis of apoptosis by FITC-Annexin V/PI double staining flow cytometry. All experiments were repeated at least three times. Data are presented as the means ± SD. *P < 0.05, **P < 0.01, ***P < 0.001 and ****P < 0.0001.

MTT assay was used to evaluate the *in vitro* renal protective effect of Fu-4-PBA/Po NPs, as shown in Fig. 2A. Cisplatin significantly inhibited HK-2 cell proliferation with an inhibition rate of 54.03 ± 2.84 %. Fu-4-PBA/Po NPs protected against cisplatin induced HK-2 cell proliferation inhibition, with an inhibition rate of only 19.35 ± 6.50 % for 10 μ g/mL Fu-4-PBA/Po NPs. It is worth noting that both free Po, 4-PBA, and 4-PBA/Po NPs have no effect on the proliferation of HK-2 cells at the usage concentration, demonstrating the safety of the usage concentration (Fig. 2B). Po has significant *in vitro* and *in vivo* antioxidant activity, and an increasing number of studies have shown that it plays an important role in kidney protection, especially in AKI and drug (such as cisplatin) or toxin induced kidney injury (Zhou et al., 2022a). The results of Fig. 2C-E showed that compared to free Po, the *in vitro* antioxidant activity of Fu-4-PBA/Po NPs was significantly increased. We believe that this may be attributed to the increased dispersibility of Po in solution caused by nanoparticles, which in turn enhances its antioxidant activity, consistent with previous studies.(Wang et al., 2025a) We further investigated the effect of Fu-4-PBA/Po NPs on reactive oxygen species (ROS). The results showed (Fig. 2F-2H) that cisplatin significantly induced the accumulation of ROS in HK-2 cells. Although free Po and Fu-4-PBA/Po NPs both inhibited cisplatin induced ROS accumulation, the inhibitory effect of Fu-4-PBA/Po NPs was more significant.

The Annexin V-FITC/PI results showed that compared with the control group, cisplatin significantly induced apoptosis in HK-2 cells, while Fu-4-PBA/Po NPs inhibited cisplatin induced apoptosis (Fig. 2I and 2J). The above results confirmed the protective effect of Fu-4-PBA/Po NPs on cisplatin induced HK-2 cell damage *in vitro*.

### 3.3 Dual targeting characteristics of Fu-4-PBA/Po NPs

**Fig. 3:**
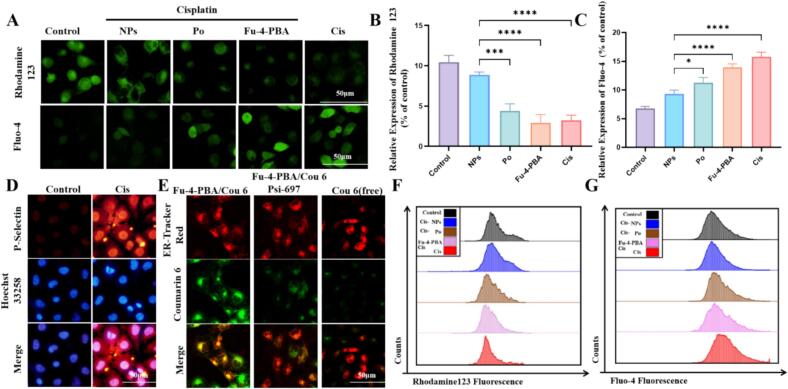
(A, B, C) Mitochondrial membrane potential and intracellular calcium ion concentration on HK-2 cells were detected by Rhodamine 123 and Fluo-4 staining. (D) Validation of increased P-selectin expression on HUVEC cells at inflammatory sites. (E) Validation of the co-localization of the endoplasmic reticulum with NPs and the uptake of coumarin 6 on HK-2 cells. (F, G) Detection of mitochondrial membrane potential and intracellular calcium ion concentration on HK-2 cells by Rhodamine 123 and Fluo-4 staining combined with flow cytometry. All experiments were repeated at least three times. Data are presented as the means ± SD. *P < 0.05, **P < 0.01, ***P < 0.001 and ****P < 0.0001.

ROS, including superoxide anion (O₂·^−^), hydrogen peroxide (H₂O₂), and hydroxyl radical (·OH), are byproducts of mitochondrial electron transport chain (ETC) activity.(Pham et al., 2024) A decline in mitochondrial membrane potential (*ΔΨm*) disrupts ETC efficiency, leading to electron leakage and increased ROS production. Furthermore, excessive ROS induces mitochondrial permeability transition pore (mPTP) opening, resulting in *ΔΨm* collapse and apoptosis. The Rhodamine 123 results showed that cisplatin treatment led to *ΔΨm* disruption and was inhibited by Fu-4-PBA/Po NPs (Fig. 3 A and 3B). Cell stress and injury may result in the accumulation of unfolded or misfolded protein, which triggers ERS. More and more studies have confirmed the important role of ERS in cisplatin induced AKI.(Li et al., 2022) The ER is an important reservoir of calcium ions in cells, and Ca^2+^ are released into the cytoplasm during endoplasmic reticulum transport (Walter and Ron, 2011). The Fluo-4 staining results showed that cisplatin treatment significantly increased the intracellular Ca^2+^ concentration, indicating the occurrence of endoplasmic reticulum stress. Interestingly, Fu-4-PBA/Po NPs alleviated cisplatin induced Ca^2+^ release (Fig. 3 A and 3C). Moreover, flow cytometry results also showed that cisplatin induced ROS accumulation and Ca^2+^ release were inhibited by Fu-4-PBA/Po NPs (Fig. 3F and 3G).

P-Selectin is a cell adhesion molecule stored in endothelial cells. Under inflammation or injury stimulation, P-selectin rapidly expresses on the cell surface to mediate inflammatory responses. Fu has been confirmed to target P-selectin, providing a new option for developing nano drug delivery based on P-selectin targeting. We cultured human umbilical vein endothelial cells (HUVEC) in the supernatant of HK-2 cells stimulated with cisplatin to simulate the expression of P-selectin at the site of inflammation. Immunofluorescence results showed that cisplatin treatment led to an increase in p-selectin expression in HUVEC cells (Fig. 3D). Furthermore, we constructed Fu-4-PBA/Cou6 NPs loaded with coumarin 6 (Cou6) to observe their targeting properties. Fu-4-PBA/Cou6 NPs exhibited similar physicochemical properties to Fu-4-PBA/Po NPs (Fig. S4). In addition, we used a Caco-2 cell monolayer model to simulate the intestinal barrier and assess the transmembrane transport ability of the NPs (Fig. S5). The results (Fig. 3E) showed that Fu-4-PBA/Cou6 NPs could be localized in the ER, while the localization of Fu-4-PBA/Cou6 NPs in the ER was reduced after treatment with Psi-697 (p-selectin inhibitor). This result is exciting, as Fu mediates the targeting of NPs to inflammatory sites through the targeting of p-selectin, and further 4-PBA mediates the localization of NPs to the endoplasmic reticulum, resulting in NPs with “dual targeting” characteristics.

### 3.4 Fu-4-PBA/Po NPs alleviating cisplatin induced ERS

Emerging research has increasingly highlighted the critical role of ER dysfunction in kidney diseases.(Jin et al., 2024) Under stress conditions, the accumulation of unfolded or misfolded proteins triggers the dissociation of glucose-regulated protein 78 (GRP78, also known as binding immunoglobulin protein, BiP), leading to the activation of the unfolded protein response (UPR). Under ER stress, the chaperone GRP78/BiP dissociates from PERK, leading to PERK dimerization. The activated PERK phosphorylates eukaryotic initiation factor 2α (eIF2α) and promotes the translation of activating transcription factor 4 (ATF4). Persistent ER stress upregulates pro-apoptotic factors such as CCAAT/enhancer-binding protein homologous protein (CHOP), a key mediator of programmed cell death, thereby exacerbating kidney injury.(Gundu et al., 2022)

**Fig. 4:**
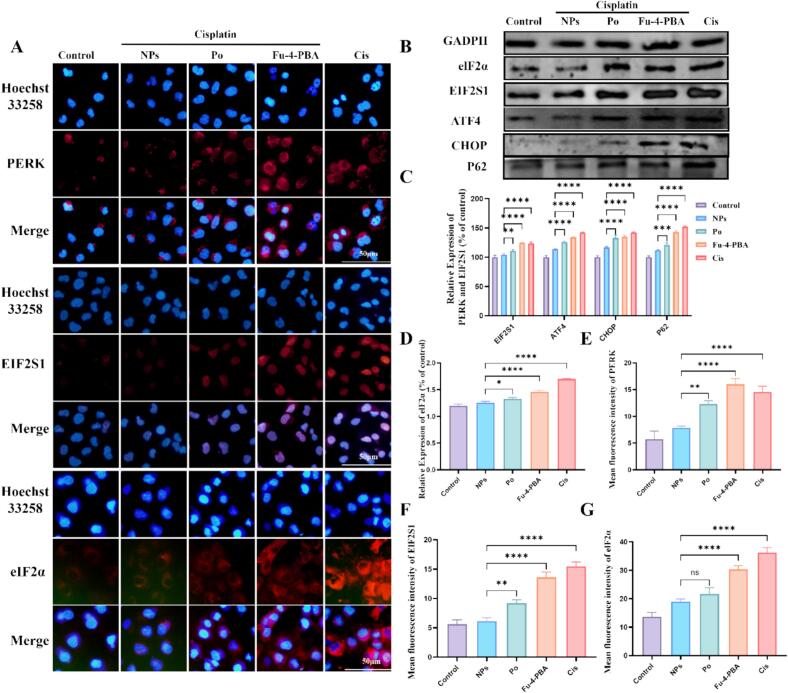
The effect of Fu-4-PBA/Po NPs on the PERK pathway. (A, E, F, G) Immunofluorescence analysis and quantitative analysis of the expression of PERK, ELF2S1, and elF2α on HK-2 cells. (B, C, D) Western blot analysis of the expression of elF2α, ELF2S1, ATF4 and CHOP on HK-2 cells. All experiments were repeated at least three times. Data are presented as the means ± SD. *P < 0.05, **P < 0.01, ***P < 0.001, and ****P < 0.0001.

We used immunofluorescence and Western blot to detect the effect of Fu-4-PBA/Po NPs on ERS mediated PERK signaling pathway. The results showed that (Fig. 4 A-G), cisplatin treatment significantly activated PERK pathway, such as PERK, EIF2S1, ATF4 and CHOP activation. Interestingly, although Po showed some protective effects, Fu-4-PBA/Po NPs had more significant inhibitory effects on the above signaling pathways. This is due to the targeting of NPs. The ER targeting feature of 4-PBA mediates the direct interaction between Po and ER to alleviate cisplatin induced ERS.

### 3.5 Fu-4-PBA/Po NPs inhibit cisplatin induced activation of cGAS/STING pathway

**Fig. 5:**
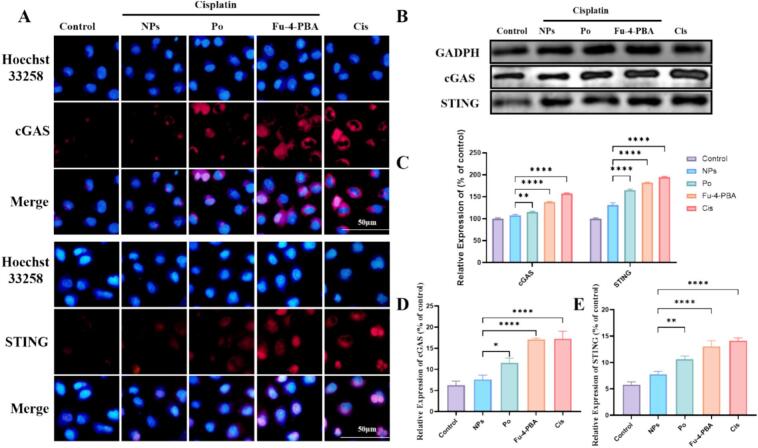
The effect of Fu-4-PBA/Po NPs on the cGAS-STING pathway. (A, D, E) Immunofluorescence analysis and quantitative analysis of the expression of cGAS and STING on HK-2 cells. (B, C) Western blot analysis of the expression of cGAS and STING on HK-2 cells. All experiments were repeated at least three times. Data are presented as the means ± SD. *P < 0.05, **P < 0.01, ***P < 0.001, and ****P < 0.0001.

cGAS-STING pathway plays a key role in inflammation, immune response and cell death. Previous studies including ours have confirmed that cGAS-STING pathway is activated in cisplatin induced AKI.(Gao et al., 2022b; Maekawa et al., 2019) As a key DNA receptor in the innate immune system, cCGAs mainly recognizes abnormal DNA in the cytoplasm (such as pathogen DNA, mitochondrial DNA or self-DNA leakage), and then activates sting dependent immune response. cCGAs-STING pathway has become an important target for the treatment of inflammatory diseases and tumors.(Dvorkin et al., 2024; Xue et al., 2024) Recent studies have found that cGAS-STING pathway and ERS interact to affect disease progression. For example, ROS accumulation induced by mitochondrial damage may activate ERS, while excessive activation of ERS will induce apoptosis, and the resulting DNA damage will further activate the cGAS-STING pathway.(Zhou et al., 2023) Immunofluorescence results showed that cisplatin treatment increased the expression of cGAS and STING, and was inhibited by Fu-4-PBA/Po NPs (Fig. 5A, 5C and 5E). Western blot results also showed that cisplatin treatment activated the cGAS-STING pathway. Although Po could inhibit the cGAS-STING pathway activated by cisplatin, the inhibitory effect of Fu-4-PBA/Po NPs was more significant (Fig. 5B and 5C).

**Fig. 6:**
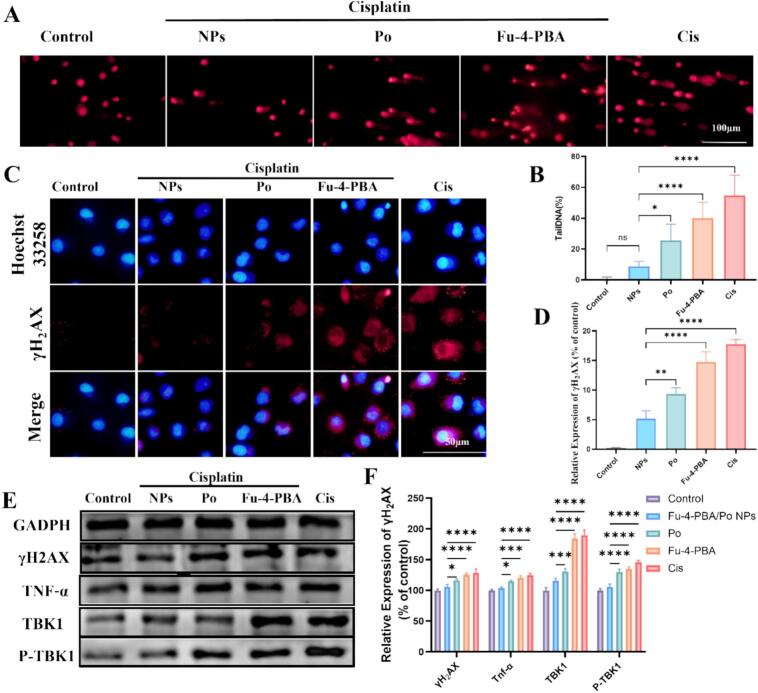
Validation of the protective effect of Fu-4-PBA/Po NPs against DNA damage on HK-2 cells. (A, B) Single-cell gel electrophoresis (comet assay) to detect DNA damage. (C, D) Immunofluorescence analysis and quantitative analysis of γH2AX expression. (E, F) Western blot analysis of the expression of γH2AX, TNF-α, TBK1, P-TBK1 and NF-κB. All experiments were repeated at least three times. Data are presented as the means ± SD. *P < 0.05, **P < 0.01, ***P < 0.001, and ****P < 0.0001.

In cisplatin treated renal tubular cells, it causes DNA damage and release, where it activates the cGAS-STING pathway and drives inflammatory responses. We further explored the activation mechanism of cGAS, and the comet assay results showed that cisplatin induced DNA damage in HK-2 cells, exhibiting a distinct comet like pattern. Fu-4-PBA/Po NPs significantly inhibited cisplatin induced DNA fragmentation (Fig. 6A and 6B). Furthermore, we used immunofluorescence and Western blot to detect the DNA damage marker γ-H2AX, which was consistent with the comet assay results (Fig. 6C-6E). Cisplatin induced DNA double strand damage was inhibited by Fu-4-PBA/Po NPs. Western blot results showed that cisplatin induced an increase in the expression of downstream cGAS-STING molecules such as TKB and TNF-α, which were inhibited by Fu-4-PBA/Po NPs (Fig. 6E and 6F). The above results confirmed that the cGAS-STING pathway was also activated in cisplatin induced AKI, and Fu/Po NPs can exert renal protection by inhibiting this pathway. The above results provide research targets for alleviating cisplatin induced AKI, but our study still has limitations. The interaction between endoplasmic reticulum stress and the cGAS-STING pathway has not been elucidated yet. We believe that the interaction between the two is important and will provide new insights for future research.

### 3.6 *In vivo* imaging and pharmacokinetics analysis

**Fig. 7:**
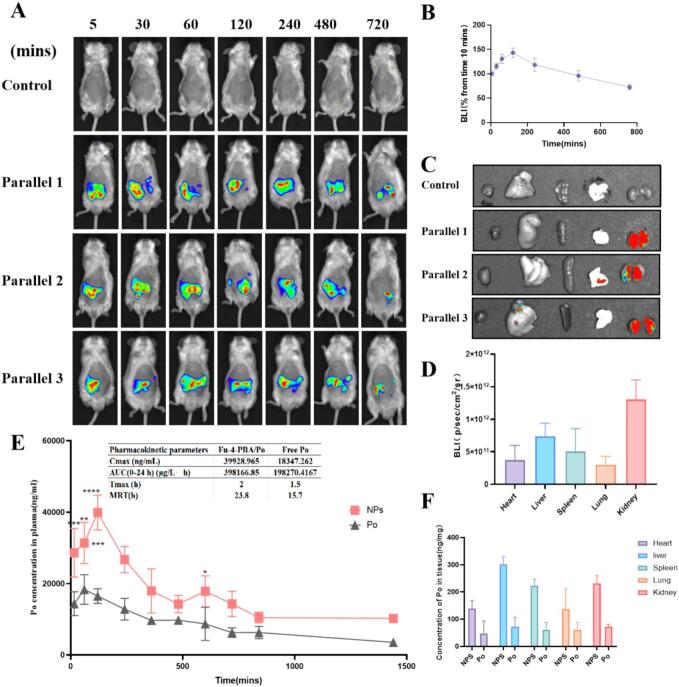
Biological distribution of Fu-4-PBA DIR in mice after oral administration for 0 to 12 h. (A) *In vivo* imaging of mice (Parelle 1–3 represents three independent replicates). (B) Comparison of abdominal luminescence intensity values of mice within 7 time points (based on 5 min). (C) *Ex vivo* images of mouse main tissues (heart, liver, spleen, lungs, kidneys). (D) Luminescence intensity values of mouse main tissues. The values are expressed as mean ± standard deviation (*n* = 3). (E) Plasma concentration–time profiles of Po after orally administration with Fu-4-PBA/Po NPs (100 mg/kg) or free Po (100 mg/kg). (B) Absorption of Fu-4-PBA/Po NPs and free Po in major organs of rats. Data are presented as mean ± SD (n = 3). *P < 0.05, **P < 0.01, ***P < 0.001 and ****P < 0.0001.

Fu-4-PBA/polydatin-DIR nanoparticles were prepared for *in vivo* imaging (Fig. S6). The results showed that the oral Fu-4-PBA delivery system mainly accumulates in the kidneys, which were the main target organs. These results were exciting, indicating that Fu-4-PBA nanoparticles have potential kidney targeting properties (Fig. 7A-7C). Fig. 7A showed the real-time biodistribution process at an early stage, while Fig. 7C provided clear evidence of the final targeted accumulation after the process is complete. To further clarify the distribution characteristics of fluorescence signals *in vivo*, the study systematically compared the *in vivo* distribution behaviors of free DIR dye and Fu-4-PBA/polydatin-DIR nanoparticles. In the free DIR dye group, the fluorescence signal rapidly dispersed after injection and reached a peak at about 45 min (Fig. S7), then declined rapidly. In contrast, the signal in the nanoparticle group decreased significantly more slowly (Fig. 7A). Together, they depict a dynamic process from initial circulation to final renal targeting. However, it must be pointed out that our research is still preliminary and more studies are needed to confirm its kidney targeting.

It is worth noting that pharmacokinetic results showed that Fu-4-PBA/Po NPs have better pharmacokinetic characteristics compared to free Po, with the peak concentration (C_max_) being 2.2 times, the area under the curve (AUC) was 2 times higher than free Po. Tissue distributions results showed that oral administration through the Fu-4-PBA delivery system mainly accumulates in the liver. In addition, the distribution of drugs in the kidneys is also encouraging. These results seem to be slightly different from the *in vivo* imaging results, and we believe that possible reasons include species differences. *In vivo* imaging is conducted in mice, while pharmacokinetics analysis is conducted in rats. The sampling time and tissue distribution sampling time for *in vivo* imaging are not consistent, which may result in slight differences in the results. The above results at least demonstrate the distribution and renal targeting potential of Fu-4-PBA *in vivo*.

### 3.7 Fu-4-PBA/Po NPs alleviate cisplatin induced AKI *in vivo*

**Fig. 8:**
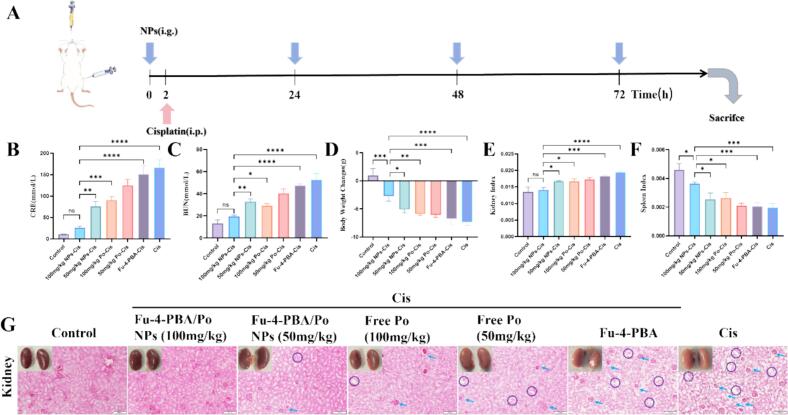
The kidney protective effect of Fu-4-PBA/Po NPs *in vivo*. (A) Schematic Diagram of Oral Administration and Cisplatin-Induced Kidney Injury Experiment in Mice. (B,C) Serum creatinine (SCr) and blood urea nitrogen (BUN) levels. (D) Body weight changes. (E,F) Kidney and spleen index. (G) Observation of histological changes in kidney tissues by hematoxylin and eosin staining (H&E), bar = 100 μm. (Blue arrows indicate inflammatory cell infiltration, while purple circles represent vacuoles in the renal tubules). Each group contained six mice (*n* = 6 biological replicates), with data representing independent measurements from individual animals. Data are presented as the means ± SD. *P < 0.05, **P < 0.01, ***P < 0.001 and ****P < 0.0001. (For interpretation of the references to colour in this figure legend, the reader is referred to the web version of this article.)

Mice were fasted and subjected to cisplatin-induced AKI *via* a single intraperitoneal injection (20 mg/kg) (Qi et al., 2023). Treatment groups received oral administration of free Po (50 mg/kg and 100 mg/kg) or Fu-4-PBA/Po NPs (50 mg/kg and 100 mg/kg) 2 h prior to cisplatin injection, followed by four consecutive days of treatment before euthanasia (Fig. 8A). Cisplatin-treated mice exhibited significant kidney pathology, including kidney whitening, edema, significant weight loss, and abnormal kidney and spleen indices (Fig. 8D-8F). In contrast, both Po and Fu-4-PBA/Po NPs treatments mitigated these pathological changes, with Fu-4-PBA/Po NPs demonstrating superior renoprotection. Consistent with morphological observations, cisplatin administration significantly elevated serum creatinine (SCr) and blood urea nitrogen (BUN) levels (Fig. 8B, 8C). Fu-4-PBA/Po NPs (100 mg/kg) effectively reduced SCr and BUN levels by approximately 84.3 % and 62.4 %, respectively, compared to the cisplatin model group (*p* < 0.001). Moreover, the nanoformulation was about 1.6-fold more effective than free Po at the same dose in lowering these biomarkers.

H&*E*-stained kidney sections revealed severe cisplatin-induced damage, characterized by inflammatory cell infiltration and tubular vacuolization. Po (50 mg/kg and 100 mg/kg) and Fu-4-PBA/Po NPs (50 mg/kg and 100 mg/kg) treatments markedly attenuated these histopathological alterations, with Fu-4-PBA/Po NPs (100 mg/kg) exhibiting the most pronounced protective effect. Collectively, these findings demonstrated that Fu-4-PBA/Po NPs significantly alleviate cisplatin-induced nephrotoxicity, surpassing the efficacy of free Po in preserving renal structure and function (Fig. 8G). Interestingly, at the therapeutic dose, no pathological changes were observed in the visceral organs of mice in each group, confirming the safety of the treatment group (Fig. S8).

### 3.8 Fu-4-PBA/Po inhibit cisplatin induced ERS *in vivo*

**Fig. 9:**
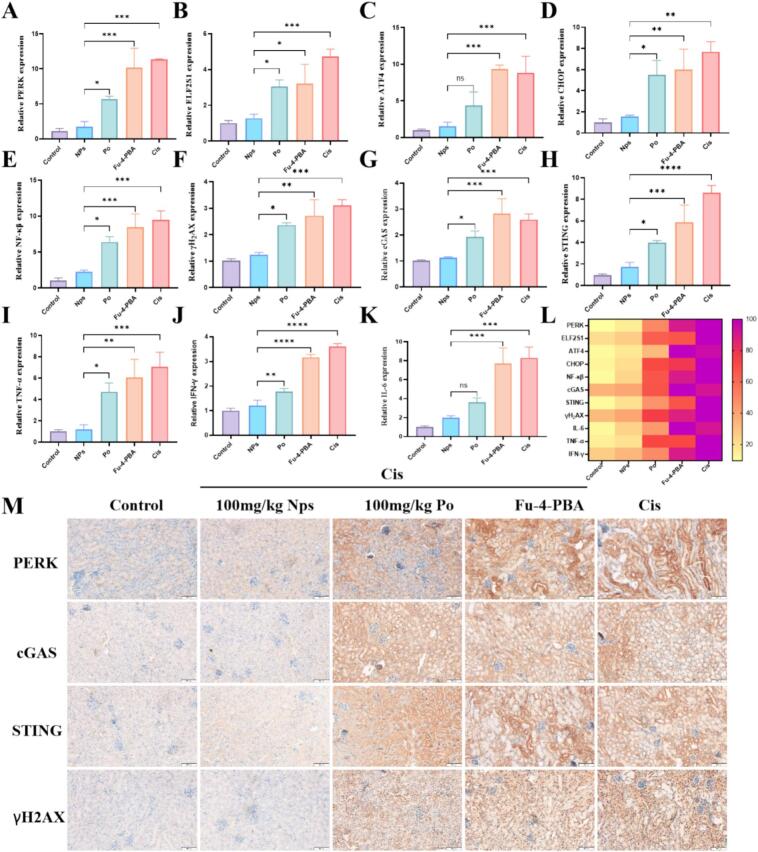
(A-E) mRNA levels of PERK, ELF2S1, ATF4, CHOP, NF-kB (F) mRNA levels of γH2AX. (G-H) mRNA levels of cGAS and STING. (I—K) mRNA levels of inflammatory cytokine factors. (L) Heatmap of PCR Factors. (M) Immunohistochemical analysis of the effect of Fu-4-PBA/Po NPs on the expression of PERK, cGAS, STING and γH2AX. Bar = 100 μm. Data are presented as the means ± SD (*n* = 3). *P < 0.05, **P < 0.01, ***P < 0.001 and ****P < 0.0001.

Recent studies have shown that multiple signaling pathways and factors contribute to the development of cisplatin induced AKI. Apoptosis of kidney tubular epithelial cells is the core pathological process of AKI.(Linkermann et al., 2014) Mitochondrial dysfunction activates the transcription of pro apoptotic genes (such as CHOP), leading to energy consumption and excessive production of ROS, resulting in cellular stress/damage, and ultimately leading to ERS-induced cell apoptosis. Therefore, AKI is attributed to mitochondrial dysfunction and ERS in the pathogen.(Yamamoto et al., 2024) The RT-qPCR results showed that the expression of PERK, ElF2S1, ATF4, and CHOP was increased in the cisplatin treated group, indicating the occurrence of ERS (Fig. 9 A-9D). Free Po showed a certain alleviating effect on cisplatin induced ERS, and interestingly, Fu-4-PBA/Po NPs significantly inhibited the expression of the ERS pathway proteins. The targeting ability of Fu-4-PBA/Po NPs plays a crucial role, as Fu can target the P-selectin at the site of inflammation, while 4-PBA further targets NPs to the endoplasmic reticulum.(DuRoss et al., 2021; Wang et al., 2024) This dual targeted NPs exhibit unique biological activity.

In recent years, an increasing number of studies have shown that the innate immune system plays an important role in the occurrence of AKI. Cisplatin induced mitochondrial damage or DNA damage may lead to cytoplasmic release of mitochondrial DNA (mtDNA) or nuclear DNA, thereby activating cGAS and inducing self-inflammation in the body.(Qi et al., 2023) RT-qPCR results showed that expression of γH2AX was increased by cisplatin treatment, followed by cGAS and STING activation. And further activate downstream inflammatory factors such as TNF-α, IFNγ, and IL-6 (Fig. 9I-9 K). Although both Po and Fu-4-PBA/Po NPs treatments inhibited the expression of the aforementioned proteins, the inhibitory effect of Fu-4-PBA/Po NPs was more significant.

Immunohistochemistry was used to further investigate the kidney protective mechanism of Fu-4-PBA/Po NPs. The results showed that the expression of PERK, cGAS, STING, and γH2AX was increased in the cisplatin treated group and inhibited by Fu-4-PBA/Po NPs (Fig. 9 M). ERS and the cGAS-STING pathway engage in bidirectional regulation, primarily mediated through inter-organelle communication and stress-induced nucleic acid release (Kuang et al., 2023; West et al., 2015). ERS, particularly *via* the PERK axis, promotes mitochondrial dysfunction and mtDNA leakage into the cytoplasm, where it activates cGAS–STING signaling. Additionally, ERS can cause nuclear DNA damage and micronucleation, further enriching cytoplasmic nucleic acids and enhancing cGAS activation (Samson and Ablasser, 2022). Conversely, STING activation increases ER protein synthesis burden and disrupts calcium homeostasis, exacerbating ERS. This reciprocal reinforcement forms a positive feedback loop that amplifies chronic inflammation and fibrosis, as observed in contexts such as cisplatin-induced toxicity and renal fibrosis (Andrade-Silva et al., 2025). The above results preliminarily elucidated the *in vivo* kidney protective mechanism of Fu-4-PBA/Po NPs, namely, cisplatin treatment leads to ERS, accompanied by mitochondrial dysfunction and DNA damage, and activates the cGAS-STING pathway. Fu-4-PBA/Po NPs have ER targeting properties and can effectively alleviate ERS caused by cisplatin. However, its mechanism is not independent, and the innate immune response mediated by the cGAS-STING pathway may be involved.

The clinical management of cisplatin-induced AKI remains a major challenge. Current standard treatments—including hydration, electrolyte management, and discontinuation of the offending drug—are largely supportive and passive in nature. In recent years, numerous novel therapeutic strategies such as antioxidants, anti-inflammatory agents, stem cell therapy, and targeted nanomedicines have been actively explored. However, most of these emerging approaches are still hampered by limitations including poor targeting, low bioavailability, and challenges in clinical translation. The Fu-4-PBA/Po NPs developed in this study exhibit significant advantages through a synergistic mechanism of action, and their active targeting capabilities offer a new direction for clinical translation. This study confirms the potential of designing an ER targeted delivery system for the treatment of AKI. In the future, we will focus on the interaction between the ERS and cGAS-STING pathways, which may provide new treatments for the prevention and treatment of cisplatin induced AKI.

## 4 Conclusions

**Fig. 10:**
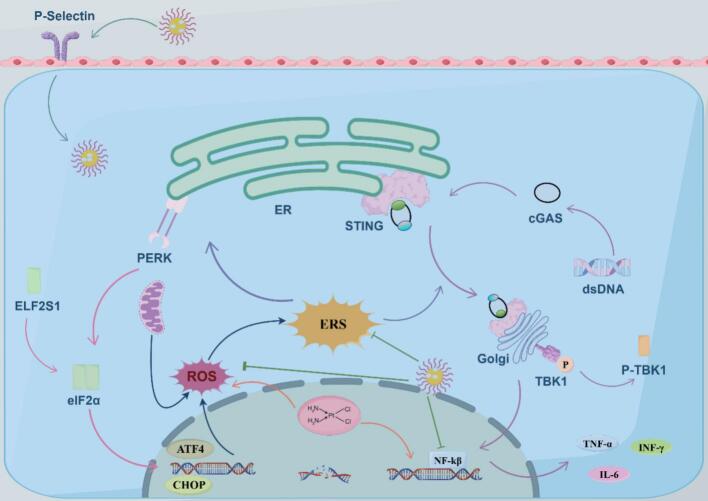
Mechanism diagram.

In this study, we modified Fu with 4-PBA and loaded Po to prepare Fu-4-PBA/Po NPs, which were used to treat cisplatin induced AKI. *In vitro* studies confirmed the dual targeting of Fu-4-PBA/Po NPs, namely the P-selectin targeting of Fu and the ER targeting of 4-PBA. The *in vitro* results confirmed that Fu-4-PBA/Po NPs significantly inhibited cisplatin induced ERS, such as the activation of the PERK-eIF2α-ATF4-CHOP pathway. Interestingly, cisplatin induced DNA damage activated the cGAS-STING pathway and was inhibited by Fu-4-PBA/Po NPs. Proving the multiplicity of the Fu-4-PBA/Po NPs (Fig. 10). *In vivo* studies also confirmed the kidney protective effect of Fu-4-PBA/Po NPs, and the protective mechanism was related to the inhibition of ERS, thanks to the excellent pharmacokinetic characteristics and ER targeting of Fu-4-PBA/Po NPs. In summary, our study provided a new perspective for the treatment of cisplatin induced AKI.

## CRediT authorship contribution statement

**Yinghan Wang:** Writing – original draft, Software, Methodology, Investigation, Data curation. **Shichao Liu:** Supervision, Resources, Investigation, Data curation. **Feikai Zhu:** Supervision, Investigation, Data curation. **Xuefei Wang:** Supervision, Methodology, Data curation. **Hanyu Wang:** Validation, Investigation. **Lin Long:** Visualization, Software, Methodology. **Jun Xiao:** Validation, Software, Funding acquisition. **Chuanlong Guo:** Writing – review & editing, Software, Funding acquisition, Formal analysis.

## Declaration of competing interest

The authors declare that they have no known competing financial interests or personal relationships that could have appeared to influence the work reported in this paper.

## Data Availability

No data was used for the research described in the article.

## Appendix A Supplementary data

Supplementary material Image 1
