# Supplementary material for: Improved bioavailability and anti-nephrotoxicity efficacy of polydatin on cisplatin-induced AKI via a dual-targeting fucoidan delivery system
Source: Int J Pharm X. 2025 Oct 18;10:100422. doi: 10.1016/j.ijpx.2025.100422 (PMC12581698; doi:10.1016/j.ijpx.2025.100422)
Supplement: Supplementary file 1 — Supplementary material [file mmc1.docx]

**Supplementary materials**

Table S1 Antibodies used in this study

| Primary Antibodies | Supplier | Catalog number | Application | Dilution |
| --- | --- | --- | --- | --- |
| γH2AX | Wuhan Sanying Biotechnology | 10856-1-AP | WB | 1:1000 |
| cGAS | Wuhan Sanying Biotechnology | 26416-1-AP | WB | 1:1000 |
| STING | Wuhan Sanying Biotechnology | 19851-1-AP | WB | 1:1000 |
| IFNγ | Wanleibio | WL02440 | WB | 1:1000 |
| TBK1 | Wuhan Sanying Biotechnology | 67211-1-lg | WB | 1:1000 |
| P-TBK1 | Beyotime | AF5959 | WB | 1:1000 |
| GAPDH | ServiceBio | GB11002 | WB | 1:1000 |
| eIF-2α | Beyotime | AF6771 | WB | 1:1000 |
| PERK | ServiceBio | GB1150507 | WB | 1:1000 |
| ELF2S1 | ServiceBio | GB11544 | WB | 1:1000 |
| elF2α | Aladdin | Ab101265 | WB | 1:1000 |
| CHOP | Immunoway | PT0105R | WB | 1:1000 |
| ATF-4 | Immunoway | PT0294R | WB | 1:1000 |
| γH2AX | Wuhan Sanying Biotechnology | 10856-1-AP | IHC-P | 1:200 |
| cGAS | Wuhan Sanying Biotechnology | 26416-1-AP | IHC-P | 1:200 |
| STING | Wuhan Sanying Biotechnology | 19851-1-AP | IHC-P | 1:200 |
| PERK | ServiceBio | GB1150507 | IHC-P | 1:200 |
| γH2AX | Wuhan Sanying Biotechnology | 10856-1-AP | IHC-P | 1:200 |
| cGAS | Wuhan Sanying Biotechnology | 26416-1-AP | IF | 1:200 |
| STING | Wuhan Sanying Biotechnology | 19851-1-AP | IF | 1:200 |
| PERK | ServiceBio | GB1150507 | IF | 1:200 |
| PERK | ServiceBio | GB1150507 | IF | 1:1000 |
| ELF2S1 | ServiceBio | GB11544 | IF | 1:1000 |
| elF2α | Aladdin | Ab101265 | IF | 1:1000 |

Table S2 primers information

| **Gene** | **Forward primer** | **Reverse primer** |
| --- | --- | --- |
| m-IFNγ | TGTTACTGCCACGGCACAGT | CTGGCTCTGCAGGATTTTCAT |
| m-P65 | CCTCCAACCCGGCGTATT | GTTTGAGATCTGCCCTGATGGTA |
| m-IL-6 | ACCACTCCCAACAGACCTGTCT | CAGATTGTTTTCTGCAAGTGCAT |
| m-TNF-α | ACAAGGCTGCCCCGACTAC | TGGGCTCATACCAGGGTTTG |
| m-H2AX | GCGGTGCTCGAGTACCTCACT | CCAGCAGCTTGTTGAGCTCCT |
| m-cGAS | AAGAGTTTCAAGAGCTGGATGCA | GGCACTCAAGAAAGAATGCTAACA |
| m-STING | GGCTGGCCTGGTCATACTACA | CCCCACAGTCCAATGGAAAG |
| m-GAPDH | GCCACCCAGAAGACTGTGGAT | GGAAGGCCATGCCAGTGA |
| m-ATF-4 | GGTGGCCAAGCACTTGAAAC | TCCATTTTCTCCAACATCCAATCT |
| m-CHOP | AGGAGGTCCTGTCCTCAGATGA | ATGTGCGTGTGACCTCTGTTG |
| m-PERK | CGACGGAGCCCGATGAC | TGAGGCTAGATGAAACCAAGGAA |
| m-Elf2S1 | AATAGCTCCACCCAGGTATGTGA | GTGACCACTTTGGGCTCCAT |


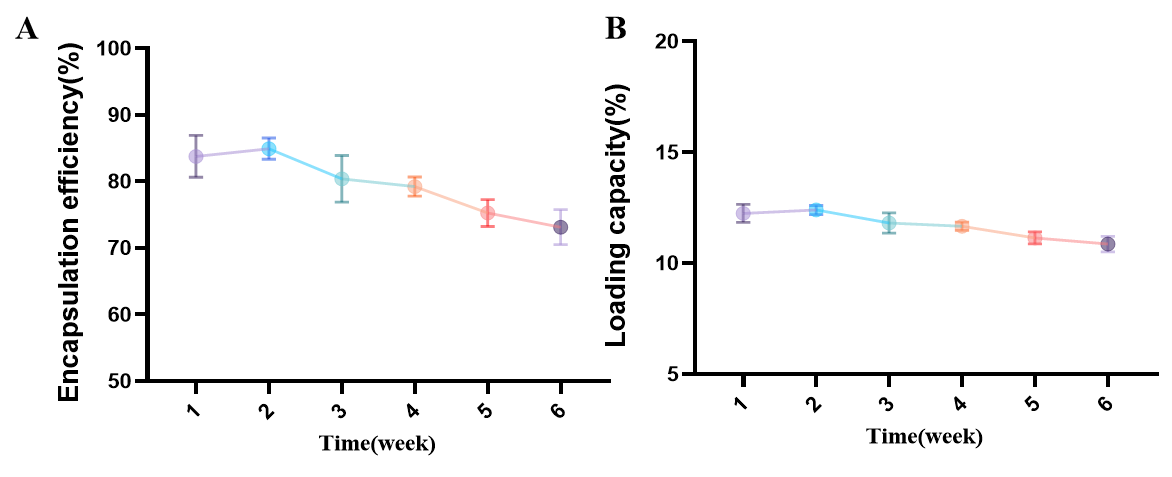


Figure S1 Stability (Cyclic) of Nanoparticles. Drug Loading (A) and Encapsulation Efficiency (B).


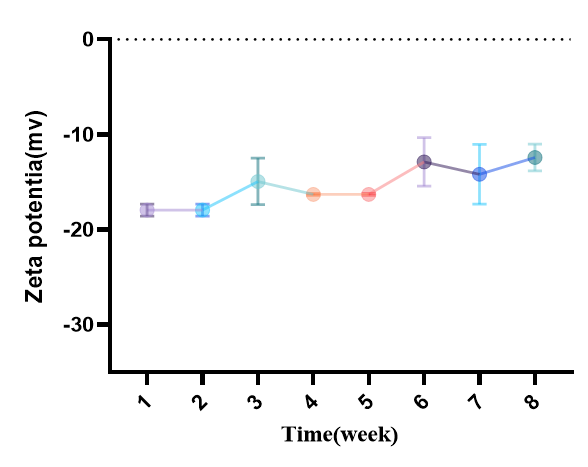


Figure S2 Zeta-potential of Fu-4-PBA/Po NPs during 8-weeks.


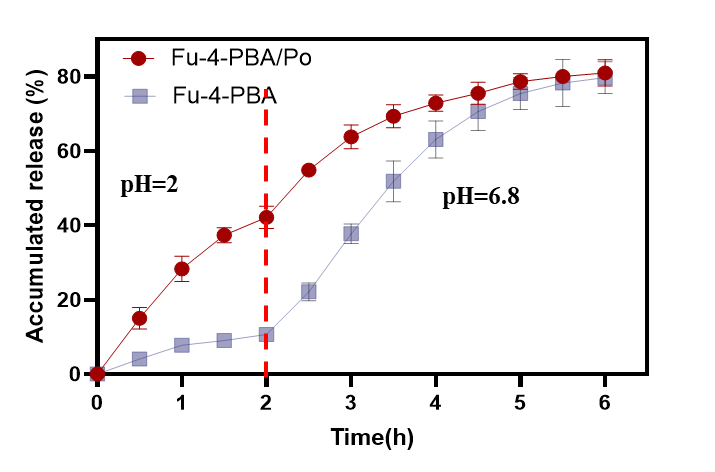


Figure S3 Release of Po and 4-PBA in Gastric and Intestinal Fluids


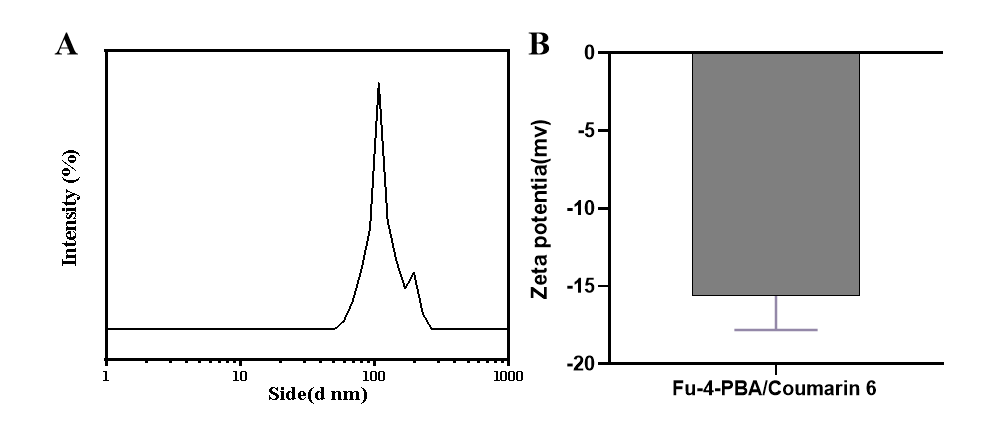


Figure S4 Particle size (A) and Zeta potential (B) of Fu-4-PBA/Cou6.


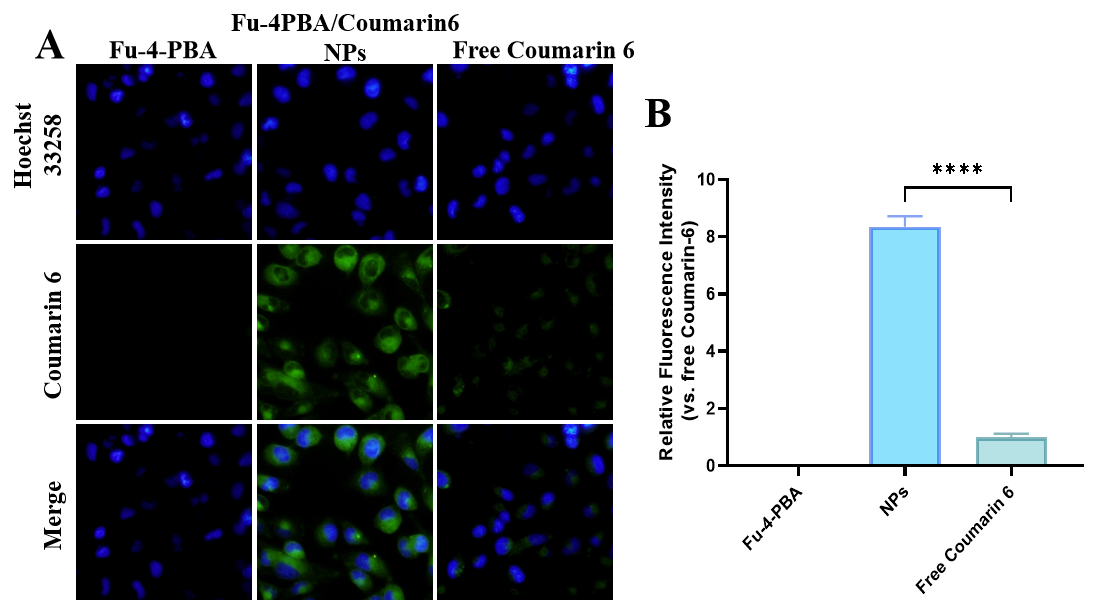


Figure S5 Evaluation of nanoparticle intestinal absorption *via* fluorescence signal amplification


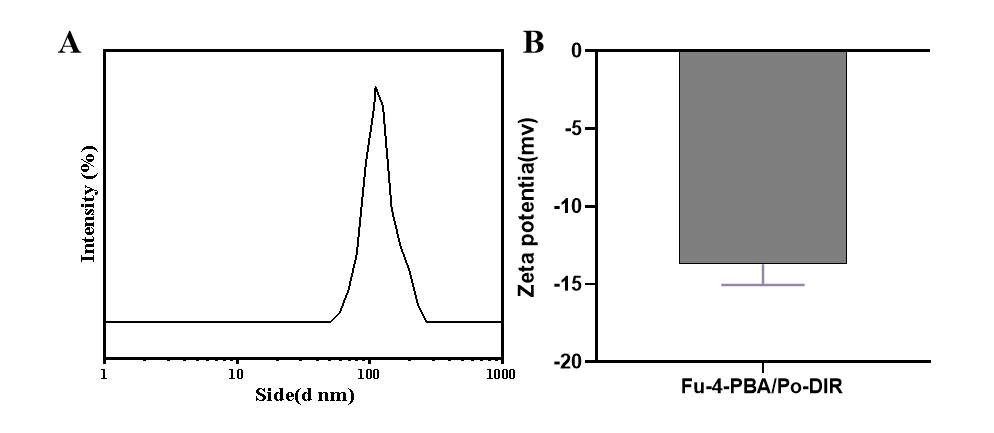
Figure S6 Particle size (A) and Zeta potential (B) of Fu-4-PBA/Po-DIR


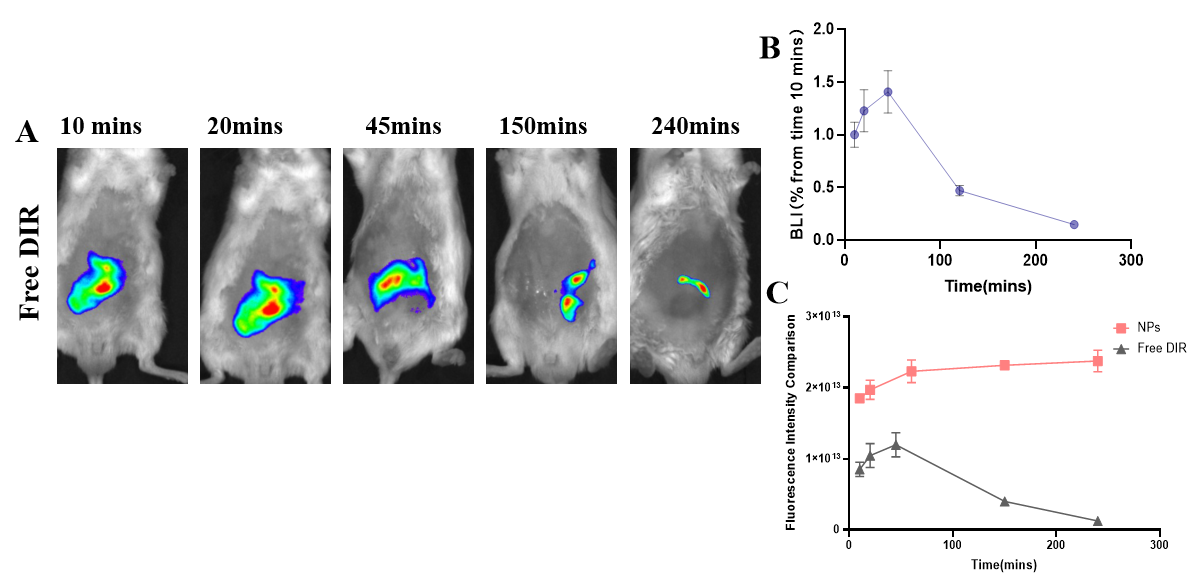


Figure S7 Fluorescence intensity of free DIR in *vivo* (A). Comparison of abdominal luminescence intensity values of mice at five time points (B). Comparison of fluorescence intensity between Fu-4-PBA/polydatin-DIR nanoparticles and free DIR(C).


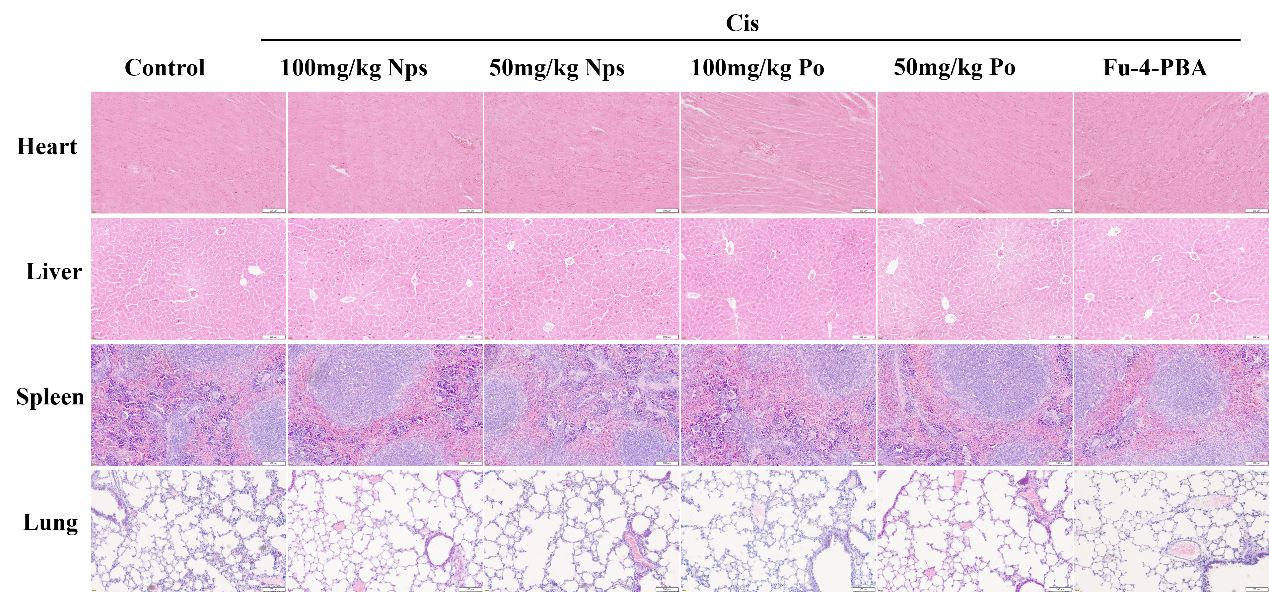


Figure S8. Safety observation of heart, liver, spleen, and lung tissues by hematoxylin and eosin staining (H&E)

**Supplementary materials-**Original Western blot images


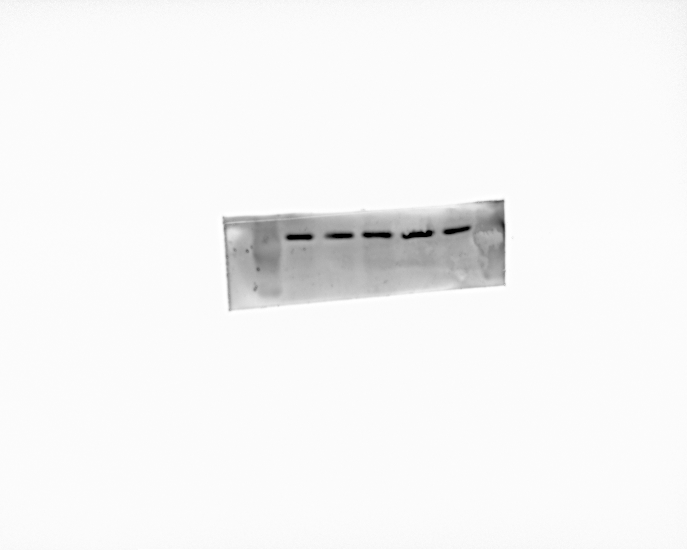


Figure 4B-GADPH


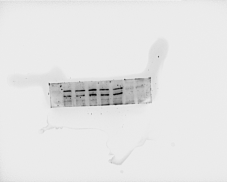


Figure 4B-elF2α


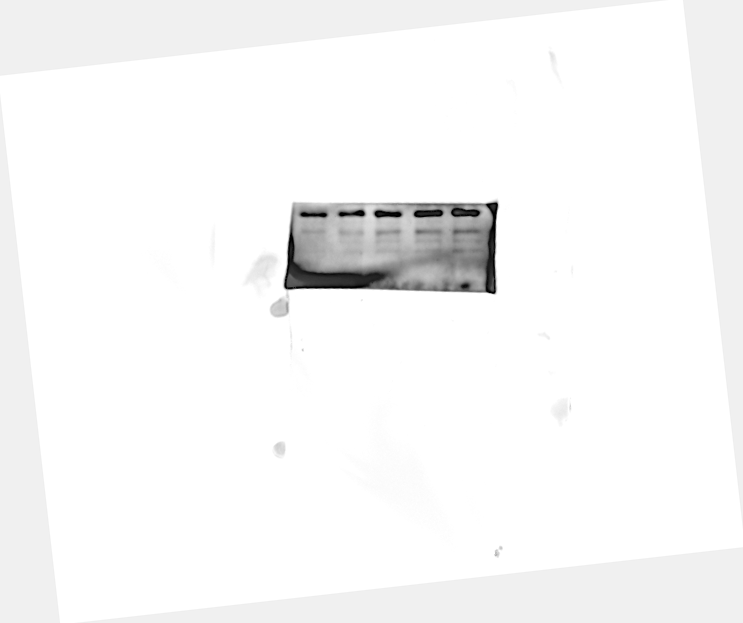


Figure 4B-ElF2S1


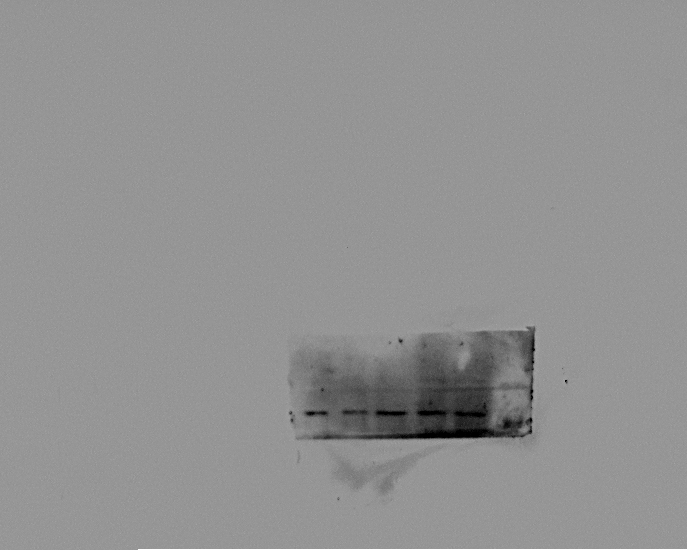


Figure 4B-ATF4


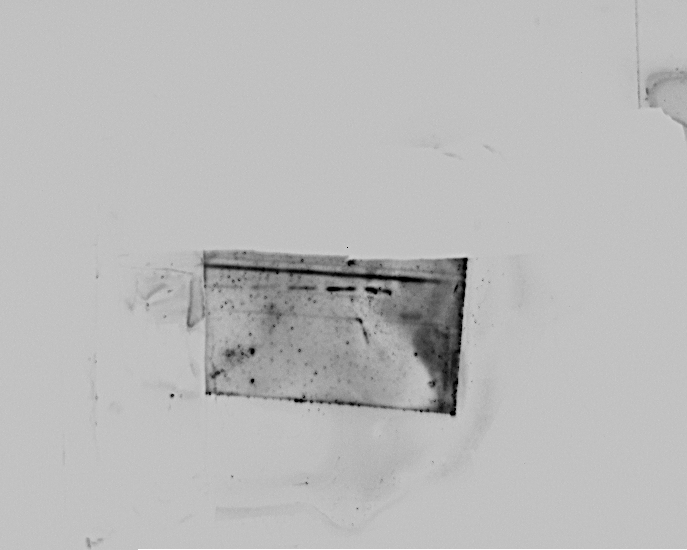


Figure 4B-CHOP


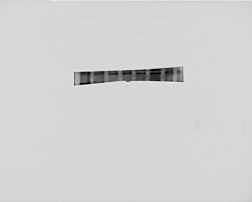


Figure 4B-p62


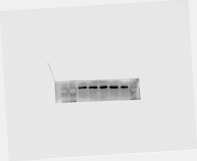


Figure 5B-GADPH


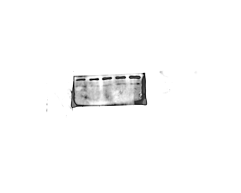


Figure 5B-cGAS


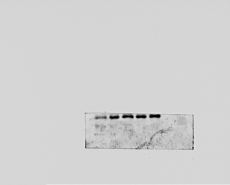


Figure 5B-STING


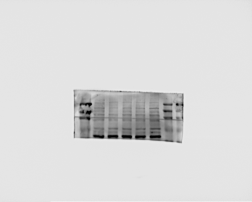


Figure 6E-GADPH


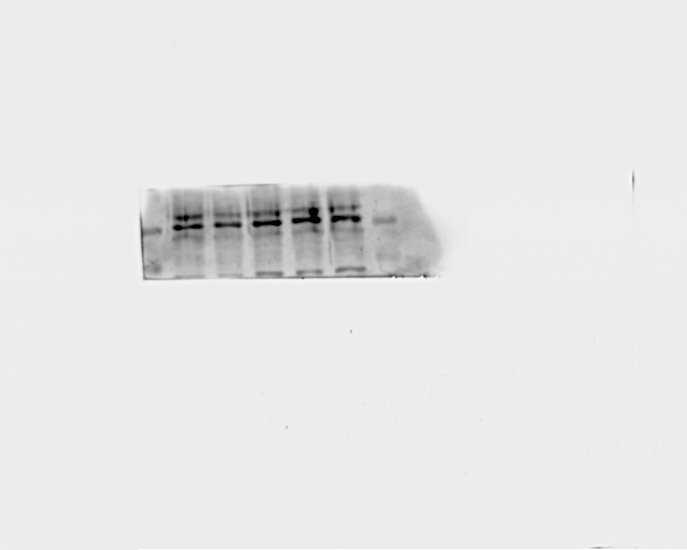


Figure 6E-γH2AX


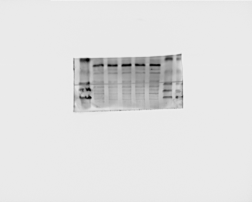


Figure 6E-TNF-α


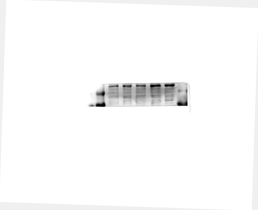


Figure 6E-TBK1


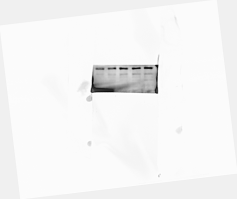


Figure 6E-p-TBK1
